# What can we learn about postnatal care in Ghana if we ask the right questions? A qualitative study

**DOI:** 10.3402/gha.v8.28515

**Published:** 2015-09-07

**Authors:** Zelee Hill, Eunice Okyere, Mary Wickenden, Charlotte Tawiah-Agyemang

**Affiliations:** 1 Institute for Global Health, University College London, London, UK; 2 Kintampo Health Research Center, Kintampo, Ghana

**Keywords:** postnatal care, indicators, household survey, validity, pre-testing, measurement, Ghana

## Abstract

**Background:**

There are increasing efforts to monitor progress in maternal and neonatal care, with household surveys the main mode of data collection. Postnatal care (PNC) is considered a priority indicator yet few countries report on it, and the need to improve the construct validity associated with PNC questions is recognized.

**Objectives:**

To determine women's knowledge of what happens to the baby after delivery, women's comprehension of terms and question phrasing related to PNC, and issues with recall periods.

**Design:**

Forty qualitative interviews and four focus group discussions were conducted with mothers, and 10 interviews with health workers in rural Ghana. Data were collected on knowledge and recall of postnatal health checks and language used to describe these health checks.

**Results:**

Mothers required specific probing using appropriate language to report postnatal checks. They only had adequate knowledge of postnatal checks, which were easily observed or required asking them a question. Respondents reported that health workers rarely communicated with mothers about what they were doing, and most women did not know the purpose of the equipment used during health checks, such as why a thermometer was being used. Knowledge of neonatal checks in the first hours after a facility delivery was low if the mother and child were separated, or if the mother was tired or weak. Many women reported that they could remember events clearly, but long recall periods affected reporting for some, especially those who had multiple checks or for those with no problems.

**Conclusions:**

Direct questions about PNC or health checks are likely to underestimate coverage. Validity of inferences can be enhanced by using appropriate verbal probes during surveys on commonly performed checks that are clear and visible to the mother.

There are increasing efforts to monitoring progress in maternal, newborn, and child health in low-income countries (1, 2). Such data help ensure accountability, garner action, identify coverage gaps, and help policy makers and implementers plan and prioritise. Current weaknesses in Health Information Management Systems include incomplete and irregular reporting, data transmission, and compilation of indicators. Given these weaknesses, nationally representative household surveys are the best way to collect data (3, 4). The Demographic and Health Surveys (DHS) and the Multiple Indicator Cluster (MIC) Surveys are the most utilized surveys and play a key role in establishing national targets, targeting interventions, and for advocacy purposes; data quality is thus paramount (4). Key issues in data quality are the ability of survey questions to garner valid responses, interview length, response rates, and sampling issues (4). The validity of responses is influenced by whether respondents understand the question in the way it is intended, whether they can provide accurate answers, and whether they are willing/able to report the answer.

The majority of maternal and neonatal deaths are in the first few days after delivery (5), and postnatal care (PNC) in the first 48 h is considered a critical intervention to reduce maternal and neonatal morbidity and mortality (6–9). Current World Health Organization (WHO) recommendations are that those who deliver in a facility receive PNC in the facility for the first 24 h, and those who deliver at home receive a home visit within 24 h of delivery. Subsequently, three home visits are recommended on day 3, between day 7–14 and at 6 weeks (6). The recommended content of PNC is shown in Table 1.

**Table 1:** WHO recommendations on postnatal care of mother and newborn

	Mother	Baby
In first 24 h if delivered in a facility	Regular assessment of vaginal bleeding, uterine contraction, fundal height, temperature, pulse. Blood pressure shortly after birth and within 6 h. Urine void documented within 6 h	A full clinical examination around 1 h after birth and before discharge
At subsequent visits, enquire	General well-being, emotional well-being, family support, resumption of sexual intercourse, and possible dyspareunia	
At subsequent visits, assess and refer if needed	Micturition and urinary incontinence, bowel function, healing of any perineal wound, headache, fatigue, back pain, perineal pain and perineal hygiene, breast pain, uterine tenderness and lochia, breastfeeding progress	Stopped feeding well, history of convulsions, fast breathing, severe chest in-drawing, no spontaneous movement, fever, low body temperature, jaundice in first 24 h
	Observe for signs or risk of domestic abuse	Identify low birth weight and provide special care
At subsequent visits, counsel	Danger signs and care seeking, nutrition, hygiene, family planning, safe sex, use of treated bednets for mother and baby (malaria areas), gentle exercise, rest	Danger signs and care seeking, exclusive breastfeeding, cord care, thermal care, immunization, play

Adapted from Ref. (6).

PNC is considered a priority indicator by the Accountability Commission for Health of Women and Children, and by Countdown to 2015, which tracks coverage levels for health interventions (1, 2). Both the DHS and the MIC Surveys have questions on PNC that ask about ‘checks on health’ for the mother and child. Both surveys explain checks to participants as examining/assessing and asking questions about health (10, 11). Despite this, only 32 of the 75 Countdown to 2015 countries reported on PNC in the first 2 days for the mother, and only 17 on PNC for the baby (12). In addition to the low measurement coverage, there are also validity concerns related to PNC questions. These included whether mothers know what happens after delivery, can recall events from several years ago, and understand questions on PNC (13). We found two studies that compared maternal reports on PNC content with observations (Mozambique) (3), or with records (China) (14). The China study reported that indicators on PNC contacts and content had moderate validity, and the Mozambique study reported that indicators on the coverage of blood pressure being taken after delivery had unacceptable validity based on area under the receiver-operating curve (3, 14). This suggests that there is room to improve questions on this area.

With the aim of understanding how construct validity associated with items on PNC questionnaires and surveys can be improved, we conducted qualitative work in Ghana to determine women's knowledge of what happens to the baby after delivery, women's comprehension of terms and question phrasing related to postnatal checks, and issues with recall periods. The PNC policy in Ghana is to promote delivery and immediate post-partum care with a skilled healthcare provider, followed by two visits in the first week of life to counsel on healthy behaviours and assess for danger signs in the mother and child (15). DHS data report coverage of PNC within 2 days of delivery as 67% (16).

## Materials and methods

### Human subjects approval

The study received ethical clearance from the committees of the London School of Hygiene and Tropical Medicine and Kintampo Health Research Center. Informed consent was obtained from all participants.

### Setting

The study was conducted in three districts in the Brong Ahafo region of Ghana over a 2-month period in 2010. The districts were chosen as they were part of a randomized control trial, ‘Newhints’, testing the impact of home visits by community volunteers on neonatal mortality (17, 18). The Newhints study was not linked to the study reported here but included a monthly demographic and health surveillance system, which was used to identify respondents for this study. The districts were predominantly rural, multi-ethnic, and education levels were low. The main occupation was subsistence farming, and few villages were reached by paved roads. Each district had a hospital staffed by a clinical officer and medical assistants and a series of health posts. At the time of the study, 65% of births were in a health facility, and nearly all pregnant women attended antenatal care at least once (unpublished surveillance data). The Brong Ahafo region is fairly typical of rural Ghana and compared to other regions’ ranks in the middle in terms of literacy, total fertility rate, levels of facility delivery, and PNC coverage. PNC coverage ranges from 49 to 88% across Ghana's regions, with coverage in Brong Ahafo of 72% (16).

### Development of interview guides

The interview guides were developed by the authors. These guides were piloted, first with the interviewers, and then with three recently delivered women to ensure that the interview length and structure were appropriate.

### Interview methods, content, and sample size

Narrative data on PNC were collected during 40 narrative interviews and four focus group discussions (FGDs) with 6–10 mothers, and 10 in-depth interviews with health workers. The aim of each method is shown in Table 2, with narratives used to allow the detailed exploration of personal experiences and FGDs to determine the language used to talk about PNC as this would be enhanced through peer discussion. The narrative interviews started by asking mothers to narrate what happened in the first 24 h after delivery in as much detail as possible; mothers were then asked about contacts with health workers in the first month of life and what happened in these contacts, followed by open questions on any ‘checks’ on themselves or the baby. Finally, structured probes were used to enquire about maternal checks related to bleeding, temperature, pulse, weight, breasts, and abdomen; and newborn checks related to temperature, breathing, cord, skin, weight, and feeding. The weight of the newborn was verified by checking the maternal record card that is given to mothers after delivery. The FGDs started by asking mothers about the meaning of the term ‘check’, this was followed by respondents’ discussing the checks women receive after delivery, and the most easy to understand way of describing these checks. Finally, the groups were asked whether mothers see what happens after delivery, and potential barriers to reporting and remembering what they saw. The health workers interviews focused on what happens to mothers and babies between delivery and discharge, what checks are done, and how these checks are explained to mothers. Health workers were also asked to define PNC.

**Table 2:** Sampling strategy and aim of each method

Method	Sampling strategy	Aim
40 PNC narratives	Mothers with children under 12 months selected from the demographic and health surveillance system to ensure a range of place of delivery, time since delivery, PNC contact with a Newhints worker and to reflect district diversity	What PNC contacts and checks occur Language used to talk about PNC Knowledge and recall of PNC and what affects these How knowledge and recall differ by place of birth and time since delivery
Four focus groups with mothers	Mothers with children under 12 months selected by community-based fieldworkers to reflect district diversity	Language used to talk about PNC Recall of PNC and what affects this
10 interviews with health workers	Selected based on sources of care described in the narratives	Health workers’ perceptions on the content and timing of PNC

Sample size was driven by the concept of saturation sampling, that is, we continued interviewing until no new information emerged. Saturation was determined through reading of all transcripts and through frequent reflection meetings to discuss emerging themes.

### Participant selection

Women for the narrative interviews were selected from a demographic surveillance system to ensure the inclusion of a range of ethnicities, education levels, delivery locations, and time since delivery. Selection also ensured that some women who had received a postnatal visit from the Newhints community volunteers (CBSVs) (see Table 3). The Newhints PNC visits included assessing the baby (including taking the baby's temperature, counting breaths, and looking for chest in-drawing), and referring any potentially sick babies to a facility. Women who participated in the FGDs were selected by community informants and were stratified by ethnic group, they were selected to include talkative women who had delivered both in facilities and at home. Health workers were selected based on the sources of care described by the narrative women. Selected respondents were approached in their home or place of work by the interviewers who explained the study, answered questions, and took consent. No participants refused.

**Table 3:** Narrative respondent characteristics

Characteristic	Number
Age (years)	
< 25	10
25–35	23
> 35	7
Education	
None	17
Primary	15
Secondary or above	8
Place of delivery	
Facility	21
Home	19
Occupation	
None	3
Farmer	24
Seamstress	5
Hairdresser	4
Trader	3
Other	1
Parity	
1	10
2–5	25
> 5	5
Time since delivery	
< 6 months	17
6–12 months	23
Received NEWHINTS PNC visit	
Yes	9
No	31

### Data collection

Data were collected by three trained fieldworkers, who conducted 1–2 interviews a day that lasted between 30 and 60 min. Data were collected in the local language (Twi) and were tape-recorded. Field notes were also taken. FGDs had both a facilitator and a note taker, who were trained to encourage group interaction. Interviews and FGDs were translated and transcribed into English by the interviewer on the day of the interview. Care was taken to preserve all relevant Twi terms and phrases. All interviews were reviewed by the study principal investigator for quality, with daily feedback given and reflective meetings held.

### Data management and analysis

Data analysis was conducted by the principal investigator, and findings were reviewed by the interviewers to check for consistency of interpretation. Analysis consisted of multiple readings of the transcripts to ensure familiarity with the data. Broad analytical categories were identified deductively based on the research questions, and data were then coded inductively based on emerging themes and indexed using Nvivo. Deductive codes included: Knowledge of health check, Interpretation of health check, Recall of health check, and Willingness to report health check. The deductive and inductive codes were then charted in excel following the framework methodology, which summarizes the key findings by respondents (rows in the framework) and by codes (columns in the framework) (19). This allowed for associations and relationships to be explored, and for data to be more easily compared and contrasted.

## Results

The results focus on data from the narrative interviews as these provided the most detailed information, with supporting data from the FGD and in-depth interviews with health workers.

Neither women who participated in the narrative interviewers nor the FGDs reported that specific people, or visits, were for ‘checking health’ after delivery. The exception was women who were visited at home as part of the Newhints trial ‘He came to “check” the children because he does it for everyone in the community when they gave birth’ (29-year-old, with no education who delivered at home 4 months ago). Health workers themselves had varied definitions of PNC, ranging from a contact at a specific time, 2 weeks to 40 days, to any care provided in the first month/6 weeks. No health workers defined postnatal care as occurring specifically in the first days after birth, and checks in the delivery facility were not considered PNC.

### Knowledge that a check had occurred

Women were rarely told what checks were being done, and communication between health staff and mothers was minimal. The major themes around factors influencing maternal knowledge of checks are shown in Table 4. Women who were interviewed knew checks had occurred when they observed equipment such as a thermometer or sphygmomanometer being used, or when someone asked them a question, for example, about bleeding, pain, or breastfeeding. For newborn checks, knowledge was influenced by where the baby was after delivery and the state of the mother. These determined whether the mother was able to observe checks.

**Table 4:** Major themes and assigned quotes around knowledge that a check occurred

Observing equipment or being asked a question
Interviewer: Did someone measure the baby's weight?
Respondent: Yes, after delivery when the nurse wiped the blood, she put him on a white ‘scale’ …. I saw that one. (28-year-old Hausa who delivered in a district hospital 6 months ago)
Assuming check done
Interviewer: After you delivered, did anybody check to see if blood was coming out or not?
Respondent: Yes. They checked it.
Interviewer: How did they check that?
Respondent: [Laughing] What should I even say? After delivery they have to make sure that blood is not coming out too much. If blood was coming from me too much they would have told me and done something about it. (22-year-old with primary education who delivered at home 3 months ago)
Being sick or tired
I didn't sleep the whole night because of the delivery and so I was feeling sleepy when the baby came out. Maybe she was doing some of the things but I didn't see because I was tired and feeling sleepy. (35-year-old with secondary education who delivered in district hospital 5 months ago)

Separation of mother and baby in home deliveries was rare, but around half of the interviewed women who delivered in the facility reported that the baby was taken out of sight to be ‘seen to’ or bathed. The baby was often taken to the maternity ward where the mother joined them when they were strong enough to walk. This meant that knowledge of whether checks, such as birth weight, were done was rare. Half of the women who had a birth weight in their maternal record reported that birth weight had not been taken.

Women who were interviewed also made assumptions about things they observed. For example, unwrapping the baby, or touching the cord, was assumed to be a cord check, and women also assumed that some checks were universally done. For example, around half of the women who reported bleeding, and most who reported a cord check, did so on the assumption that it was done rather than necessarily knowing at the time. None of the women who delivered at home reported that being sick or tired affected their knowledge of whether checks were done, but around half of those who delivered in facilities said they were unsure what happened to them or the baby in the first hours after delivery because they were tired or weak or were being attended to.

### Interpretation of the check

Although women who were interviewed were able to observe the use of equipment, most women did not know what the thermometer, stethoscope, or blood pressure cuff were for specifically, except that it was ‘seeing their health’, ‘seeing if they had any sickness’, ‘looking at their strength’, or ‘see how their body is doing’. A few women had misconceptions about what the instruments were for.

Interviewer: … did they ‘checkie’ his breathing?

Respondent: They ‘checkie’ it ….

Interviewer: What did they do that makes you think they ‘checkie’ his breathing?

Respondent: Is it not what they put at his armpit? (30-year-old Banda with primary education who delivered in a district hospital 3 months ago)

Interviewer: Did someone ‘check’ to see if your body was hot or cold?

Respondent: You mean if someone put her hand on my forehead?

Interviewer: I mean did someone put something under your armpit to see if your body is hot or cold?

Respondent: Ah, yes. The ‘nurse’ put something under my armpit and left it there ….

Interviewer: Do you know what that thing is for?

Respondent: I never knew what it was for until today … I never knew it was to check body hotness or coldness. (21-year-old with Primary education who delivered in a Health Center 9 months ago)

Over half of the women who were interviewed that reported having their temperature taken had to have the thermometer described before they gave an answer, with similar findings related to having the blood pressure cuff described. Knowledge of checks was higher amongst educated women (secondary school and above), who knew terms such as ‘BP’ and ‘temperature’. A key issue related to this lack of knowledge was a lack of communication between the mothers and the health staff. This finding was confirmed by all respondent groups:

Interviewer: So what did they say they were doing when they put it [thermometer] there?

Respondent: The nurse didn't tell me … I didn't know what she was doing.

Interviewer: So why didn't you ask her?

Respondent: Eh. I can't ask because they are doing their work. Some of the nurses don't have patience so if you go and ask her some questions … she would insult you … If they are doing something you have to keep quiet and look at them. They don't tell you anything when they come. (27-year-old with Secondary school education who delivered in a district hospital 11 months ago)

Our workload is too much unless we do a check and there is an abnormality … if that happens we tell her what the problem is. (Midwife in district hospital)

Other issues with interpretation were: reporting delivery activities such as pressing on the abdomen during delivery as a check, and difficulty differentiating between a check and being given advice or performing an activity, as illustrated by this mother:

Interviewer: Did someone check how the baby was feeding?

Respondent: Yes. Mr. X told me to give the breast milk to him often. (32-year-old with primary education who delivered at home 11 months ago)

Checks were done by a variety of people including traditional birth attendants for those who delivered at home, healers, and family members. Traditional birth attendants usually returned to the house after delivery ‘to see how you and baby are faring and whether the baby is crying …. At times they come 3 or 4 times …’ (37-year-old focus group respondent who delivered in a facility 4 months ago). Interpretation of a health worker was problematic as women did not clearly discriminate between them, considering hospital/facility workers, community health volunteers, Newhints fieldworkers, and healers all as health workers

Interviewer: Apart from the weighing people, did you see any health worker?

Respondent: Yes. We took the baby to a woman who gave us medicine to bath the baby …. She does local medicines. (36-year-old with no education who delivered in a health centre 4 months ago)

### Remembering the check

Several of the women who were interviewed that delivered more than 6 months ago said that they had problems remembering because they had delivered long ago, this was especially true if they had multiple checks:

Look at me I have almost forgotten everything. I use to remember all this initially but because it is getting longer I started forgetting everything. (42-year-old who delivered at home 6 months ago)

They did so many things on the baby but because it is almost a year now, I have forgotten most of the things they did for the baby. (32-year-old who delivered at home 11 months ago)

Women in one focus group reported that they would forget things if everything had gone well, but if there had been some problems they would remember everything that happened however long ago it happened.

Several women who were interviewed had difficulty estimating exact time periods, such as minutes or hours, but women who delivered in facilities talked with confidence about the time they arrived in the facility and when they left – often linked to the Muslims prayer times. Salient points in time for the women were: before and after the placenta was delivered, before or after they were moved from the labour to the maternity/lying in ward (occurs only in the big district hospitals and some private maternity homes), and before and after discharge.

### Willingness and ability to answer questions about checks

The idea of a health check ‘Checkie’ was well understood by women as ‘seeing if there is any sickness in the body …. See how your health is … check to see if you are strong’, and they gave examples of tests on blood, urine, and ‘toilet’ (faeces). Three words were commonly used to talk about health checks: ‘checkie’, ‘firii me’ (diagnose), and ‘hwee me’ (look at me). All FGDs reached consensus that these words can be used interchangeably, but did not agree on which is the most used or best word.

Questions phrasing was key in eliciting responses from narrative mothers, and specific probing was often required. For example, when asked if they had received any health checks in the month after delivery only eight women responded positively, but on probing 21 additional women reported a check. For some women, probes had to be very specific in terms of mentioning the check and the provider or describing the check

Interviewer: Did somebody take your body hotness?

Respondent: No.

Interviewer: But do you know how they take body hotness?

Respondent: No

Interviewer: So if they took it how would you know?

Respondent: Laughing.

Interviewer: Ok did they put anything in your armpit?

Respondent: Yes. They did it when I delivered and when I went on the 10th day they did it. (27-year-old with Secondary school education who delivered in a district hospital 11 months ago)

In addition to being linked to the level of probing, the ability to answer questions was also linked to the terminology used. This was especially for questions around blood pressure, which were best understood when terms such as ‘tied your hand’, ‘saw if your blood was high’, and ‘saw if your breathing was up’ were used.

The interviews with women who had been visited by a Newhints community volunteer indicated that there was often a strong social bond between them. A few women who were visited by the volunteer reported checks that were very unlikely given the equipment that the volunteers are provided with as part of the programme. No other issues with women being unwilling to give honest reports emerged.

## Discussion

We found that mothers required specific probing using appropriate language to report postnatal checks. They only had adequate knowledge of checks that were easily observed or required asking them a question. Health workers rarely communicated with mothers about what they were doing, and most women did not know what equipment such as a thermometer was for. Knowledge of neonatal checks in the first hours after a facility delivery was low if the mother and child were separated, or if the mother was tired or weak. Many women reported that they could remember events clearly, but long recall periods affected reporting for some, especially those who had multiple checks or for those with no problems.

Qualitative data from Bangladesh and Malawi support our findings that specific prompts need to be used when asking questions about PNC contacts (20). The recent addition to PNC questions of an introductory statement explaining checks as ‘examining’, ‘assessing’, and ‘asking questions about health’ in the DHS and the MIC Surveys (10, 11) are likely to improve valid reporting. We suggest that validity may be further improved by providing examples of checks that are commonly performed, that can be easily described, and which are clear and visible to the mother. Checks that fulfil these criteria are also candidates for signal functions, a short list of postnatal checks used in surveys to reflect the content of PNC, and five signal functions have been recommended partly informed by this study (13).

The lack of knowledge about the purpose of equipment was surprising, especially as in the most recent DHS in Ghana 97% of women reported having a blood pressure check during antenatal care (16), but in our study a theme emerged around having to have the blood pressure cuff described. Possible explanations are that the DHS translated blood pressure using descriptive terms such as ‘wrapped your hand’, which we found mothers understood. Alternatively, our finding on the need to describe equipment may not be generalizable beyond our study sample. The low knowledge of checks could be linked to health workers rarely communicating with mothers about what they were doing. Our findings support the suggestion that coverage measurement could be improved by increasing the salience of intervention delivery (21), for example, by service providers providing explanations of the interventions being delivered.

Many women reported that they could remember events clearly, but long recall periods affected reporting for a few, especially for those women who had multiple checks or where the checks did not find any problems. Findings from other studies also suggest that mothers can recall details of their delivery and care after many years (20, 22, 23).

Unlike antenatal care, PNC is not yet a well-recognized or branded concept. This is reflected in the fact that the DHS and the MIC Surveys can ask directly about antenatal care, but need to describe PNC. Recent changes in WHO guidelines (6, 9) make it likely that over the next few years home visits in the postnatal period will become more common. Our study shows that such visits quickly gain recognition as visits to check the baby, and questions on PNC may become easier to ask, and better understood, over time. This potential change in mothers’ abilities to report on PNC could affect the ability to monitor change over time.

Pre-testing has long been the standard method of improving survey questions in low-income countries and is recommended to limit error and bias (24). Pre-testing usually consists of interviewers piloting the questionnaire on a small number of respondents. Problems are identified through observing interviews, feedback from fieldworkers, and through tallying responses. For example, fieldworkers may report that when asked a certain question, respondents looked puzzled or hesitated. This method of pre-testing can mean that some problems remain unnoticed, as answers can sound reasonable but may still have been poorly understood, recalled, and reported (25). Qualitative methods, such as cognitive interviewing (26), are increasingly being used in low-income countries (27–30), but these methods focus on testing questions rather than developing them based on respondents’ descriptions of constructs and their experiences (26). We feel that using qualitative methods to develop, rather than test questions, improves validity as it allows concepts and terminologies to emerge from the respondents’ perspective and provides a better understanding of potential biases.

### Limitations

The methods we used have limitations related to the potential for recall and social desirability bias. There are also issues around generalizability, as studies on the validity of maternal and child health indicators have found high variability in sensitivity and specificity between, and within, countries; for example, between rural and urban areas (21). This variation may be linked to differences in the epidemiological context but may also be due to cultural and education differences. Our data collection was limited in the range of participants included, and selection of respondents to reflect a wider range of experiences may be merited. The themes that emerged for this population may be affected by the low coverage of postnatal visits amongst respondents and the low education levels. This study does, however, raise a range of issues that survey designers should consider when thinking about the potential for measurement error in their settings.

## Conclusions

Direct questions about PNC or health checks are likely to underestimate coverage. Recent additions to PNC questions in the DHS and the MIC Surveys are likely to improve validity. Validity may be further increased by specific questioning, and by using setting appropriate probes and examples of commonly performed health checks, that can easily be described and are clear and visible to the mother. PNC visits by community health workers appear to gain recognition and branding quickly. If these visits increase, as is recommended by the WHO, the questions about PNC may become easier to ask and answer. Improving the level of communication between health workers and their patients would increase the validity of PNC indicators and would also improve the quality of care women receive. Collecting qualitative data to improve PNC questions was extremely useful in understanding and improving the validity of PNC indicators. We recommend that survey designers should move away from relying on standard pre-testing methodologies.
